# Mitochondrial Dynamics: In Cell Reprogramming as It Is in Cancer

**DOI:** 10.1155/2017/8073721

**Published:** 2017-04-17

**Authors:** Javier Prieto, Josema Torres

**Affiliations:** ^1^Departamento Biología Celular, Biología Funcional y Antropología Física, University of Valencia, 46100 Burjassot, Spain; ^2^Instituto de Investigación Sanitaria (INCLIVA), 46010 Valencia, Spain

## Abstract

Somatic cells can be reprogrammed into a pluripotent cellular state similar to that of embryonic stem cells. Given the significant physiological differences between the somatic and pluripotent cells, cell reprogramming is associated with a profound reorganization of the somatic phenotype at all levels. The remodeling of mitochondrial morphology is one of these dramatic changes that somatic cells have to undertake during cell reprogramming. Somatic cells transform their tubular and interconnected mitochondrial network to the fragmented and isolated organelles found in pluripotent stem cells early during cell reprogramming. Accordingly, mitochondrial fission, the process whereby the mitochondria divide, plays an important role in the cell reprogramming process. Here, we present an overview of the importance of mitochondrial fission in both cell reprogramming and cellular transformation.

## 1. Introduction

Mitochondria and their movement as organelles were described for the first time 100 years ago [1]. In addition to producing energy by oxidative phosphorylation (OXPHOS) of pyruvate and beta-oxidation of lipids, the mitochondria play important roles in the regulation of a wide variety of intracellular processes, such intracellular calcium homeostasis [2], iron-sulfur protein assemblage [3], or apoptosis [4] and innate immunity cell signaling pathways [5].

There is no de novo mitochondrial biogenesis; the mitochondria divide by fission and join by fusion [6, 7]. Fission-fusion balance allows the mitochondria to acquire different structures. When fission is higher than fusion, mitochondria become fragmented and isolated. When fusion is higher than fission, these organelles display a tubular and networked morphology. Cells can shift the fission/fusion balance in response to either intracellular or extracellular stimuli. And thus, mitochondrial fission is increased during (1) G2/M phase of cell cycle, to guarantee an accurate mitochondrial segregation between the two daughter cells during cell division [8, 9]; (2) mitochondrial transport in neurons, to facilitate their transport along the axons and dendrites [10]; (3) early phase of apoptosis, to facilitate cytochrome c release into the cytoplasm by inducing mitochondrial cristae remodeling [11, 12]; or (4) mitophagy, to eliminate dysfunctional mitochondria [13]. On the other hand, mitochondrial fusion is favored during (1) G1/S transition of cell cycle, to provide with the necessary energy for DNA synthesis [14]; (2) cell survival during starvation, to maximize energy production and protect themselves against mitophagy [15, 16]; (3) mitochondrial complementation, to avert the loss of mitochondrial functions caused by damaged components of these organelles [17, 18]; or (4) embryonic development, as in trophoblast or placenta formation [19, 20]. Regulation of mitochondrial dynamics is therefore crucial for the correct implementation of mitochondrial functions. In fact, mutations in the components that drive or regulate fusion and fission processes are associated with several human pathologies, such as optic atrophy (*Opa1* gene) or Charcot-Marie-Tooth disease (*MFN2* and *GDAP1* genes) [18].

The molecular machinery that controls the fission and fusion processes includes proteins that are either localized in mitochondrial membranes or recruited to the surface of these organelles in response to different stimuli. Three key players of the fusion process are mitofusin (Mfn) 1 and 2 and optic atrophy protein 1 (Opa1), both of which are transmembrane proteins localized in the outer or inner mitochondrial membranes, respectively. Mfn1 and Mfn2 tether adjacent mitochondria by forming trans-hetero- or homocomplexes to promote the fusion of their outer membranes [17, 19]. It has been suggested that a heptad repeat region in Mfn1 adopts an antiparallel coiled coil conformation to tether neighboring mitochondria during the fusion process [21]. Cells that lack both Mfn1 and Mfn2 display fragmented mitochondria and fail in mitochondrial complementation [19, 22], which eventually leads to an accumulation of dysfunctional mitochondria [17]. Fusion of outer and inner mitochondrial membranes is a temporally linked, multistep process controlled by transmembrane adaptor proteins that span both membranes [23]. Mfn1 and Mfn2 interact with Opa1 [24], suggesting that the interaction of Mfn1/2 with Opa1 and/or other adapters physically connects both membranes to coordinate the fusion of these organelles [25]. The fission process is executed by dynamin-related protein 1 (Drp1), a cytosolic protein with GTPase activity [26, 27]. Drp1 is activated in the cytosol by posttranslational modifications in response to different stimuli and then recruited to the mitochondrial surface by its interaction with protein adapters [28, 29]. Mitochondria-recruited Drp1 oligomerizes on the external surface of mitochondria forming a ring-shaped structure around the organelle. Once a Drp1 spiral around the mitochondria is completed, the hydrolysis of GTP bound to Drp1 causes a conformational change in the protein that causes the constriction of the ring, eventually leading to the fragmentation of mitochondria in two different organelles [30, 31]. Different protein adapters for Drp1 have been described, including mitochondrial fission protein 1 (Fis1) [28], mitochondrial fission factor (Mff) [32], and mitochondrial dynamic proteins of 49 (Mid49) and 51 (Mid51) kDa [33, 34]. Recent work has shown that these Drp1 adapters could either operate together or be redundant in the recruitment of the GTPase to the mitochondria [35, 36].

**Figure 1:**
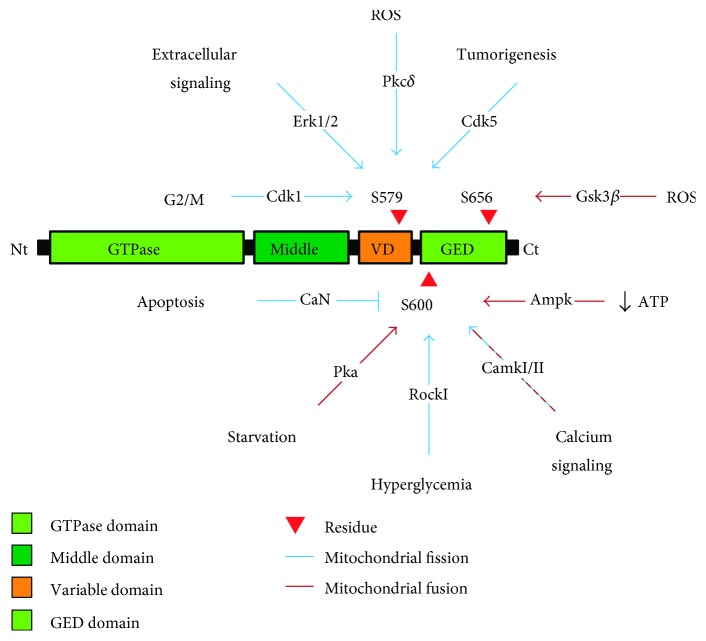
Drp1 regulation by phosphorylation. Phosphorylation of Ser579 by Cdk1 [9, 44], Pkc*δ* [45], Erk1/2 [46, 47], or Cdk5 [48] induces mitochondrial fission. Phosphorylation of Ser656 by Gsk3*β* [49] induces mitochondrial fusion. Phosphorylation of Ser600 by Pka [15, 52, 53], CamkII [48], or Ampk [55] induces mitochondrial fusion, and its dephosphorylation by calcineurin (CaN) [53, 54] induces mitochondrial fission, but, in some circumstances, phosphorylation of Ser600 by CamkI [50] or RockI [51] can induce mitochondrial fission.

Mitochondrial dynamics, in terms of the fission/fusion balance, is a highly regulated process where posttranslational modifications play a central role in the outcome of this equilibrium. Phosphorylation Mfn1 by extracellular regulated kinase 1/2 (Erk1/2) impairs its oligomerization properties and leads to decreased mitochondrial fusion [37]. Also, phosphorylation of Mfn2 by c-Jun N-terminal kinase (Jnk) results in its ubiquitin-mediated proteasomal degradation, leading to increased mitochondrial fragmentation [38]. Opa1 undergoes proteolytic processing by several proteases to produce short and long protein isoforms [39–41]; however, it is poorly understood how this proteolytic processing alters mitochondrial dynamics [42]. It is known that alterations in Opa1 proteolysis affect inner mitochondrial membrane dynamics and cristae structure [43]. Drp1 is target of several posttranslational modifications that affect its function: phosphorylation, ubiquitination, sumoylation, and nitrosylation [7]. Regarding its phosphorylation, only the phosphorylation in three Drp1 residues by different kinases has been well documented to play a role in the regulation of this protein: serine 579 (serine 616 in humans), serine 600 (serine 637 in humans), and serine 656 (serine 693 in humans). Phosphorylation of any of these three residues affects Drp1 protein-protein interactions and can either impair or favor the mitochondrial recruitment of Drp1. It has been described that Ser579 phosphorylation induces mitochondrial fission [9, 44–48]; Ser656 phosphorylation induces mitochondrial fusion [49]; and Ser600 phosphorylation induces either mitochondrial fission [50, 51] or fusion [15, 48, 52–55], depending on the cellular context (Figure 1). Recently, it has been described that AMP-activated protein kinase (Ampk) induces mitochondrial fission in response to energy stress through direct phosphorylation of Mff [56].

Endoplasmic reticulum (ER) also plays an important role during mitochondrial fission. It has been shown that ER projections wrap mitochondria around the areas where fragmentation of these organelles takes place. These ER-mitochondria contacts are not Drp1-dependent, but rather enhance the recruitment of the GTPase to these focal points [57, 58]. ER-associated inverted formin 2 (Inf2) plays an important role in mitochondrial fission by inducing the accumulation of actin filaments around the ER-mitochondria contact points. The distribution of actin filaments around the mitochondria at the ER contact points may drive an initial mitochondrial constriction to favor the action of mitochondrion-bound Drp1 [58]. Also, a profission role for ganglioside-induced differentiation-associated protein 1 (Gdap1) has been proposed, as Gdap1 favors the formation of ER-mitochondria contacts in certain neural cell types and its overexpression leads to fragmented mitochondria [59–61].

## 2. Mitochondrial Dynamics during Embryonic Development and Cell Differentiation

As the oocyte provides all the mitochondria to the zygote during fecundation, all these organelles are of maternal origin. During the first phase of embryonic development, mitochondrial biogenesis and mtDNA synthesis are not active and mitochondrial mass decays by half upon each cell division [62]. During the early stages of development, cells have a simple mitochondrial network: cristae-poor and fragmented mitochondria with low mtDNA copy number. Conversely, the mitochondrial network of somatic cells shows a complex structure: cristae-rich and tubular mitochondria with a dense mitochondrial matrix and high mtDNA copy number [62–66].

Cell differentiation during embryogenesis leads to a progressive increase in mtDNA copy number, mitochondrial mass, size, and complexity of these organelles [67–69]. For instance, the specification of cardiomyocyte [70, 71] or adipocyte [72] cell lineages is characterized by an increase in the elongation, matrix complexity, and functionality of mitochondria. Also, during cardiomyocyte differentiation, the closure of the mitochondrial permeability transition pore increases mitochondrial membrane potential and reduces reactive oxygen species (ROS) levels [71]. Mfn2 and Opa1 play an important role in this mitochondrial maturation process. Lack of *Mfn2* or *Opa1* prevents mitochondrial fusion leading to an increase in cytosolic calcium levels and calcineurin activation, which impairs efficient cardiomyocyte differentiation [73]. Mitochondrial integrity, in terms of energetics, Ca^2+^-storage/buffering, neurotransmitter metabolism, or ROS signaling, plays a central role in neuronal physiology during both development and adulthood [74]. Interestingly, a wide range of neurodegenerative diseases, such as Charcot-Marie-Tooth disease, Parkinson's disease, or several ataxias, are linked to mutations in gene-encoding proteins involved in mitochondrial dynamics, underscoring the role of this equilibrium in maintaining neuronal homeostasis [75].

The low rate of mtDNA replication observed during the very early stages of embryonic development and in embryonic stem (ES) cells is associated with high methylation levels in the genes encoding DNA mitochondrial polymerase subunit gamma [76] and mitochondrial transcription factor A (*Tfam*) [77], which impairs their expression in cells of the early embryo. However, demethylation of these genes is induced upon implantation of the embryo, leading to an increase in their expression and mtDNA replication.

Despite the profound changes experimented by the mitochondrial network during cell differentiation, the regulation of mitochondrial dynamics in ES or adult stem cells is poorly understood. ES cells present a fragmented mitochondrial morphology [65, 78]. Surprisingly, downregulation of growth factor *erv1*-like (*Gfer*) in ES cells, which leads to an increase in Drp1 protein levels and mitochondrial fission rates, impairs pluripotency and induces apoptosis [79]. On the other hand, adult neural stem (NS) cells display tubular mitochondrial morphology and NS cells derived from *Mfn1/2*- or *Opa1*-null mice, which display increased mitochondrial fragmentation, show decreased self-renewal and a greater tendency to cell commitment associated with augmented ROS and Nfe2-related factor 2 (Nrf2) expression levels [80]. These evidences strongly suggest that a proper balance of mitochondrial fission and fusion is required to maintain a homogeneous and functional mitochondrial population in the cells.

## 3. Mitochondrial Dynamics in Cell Reprogramming

Somatic cells can be reprogrammed to a pluripotent state similar to that of ES cells by ectopic expression of Oct4, Sox2, Klf4, and c-Myc (OSKM hereinafter) [81]; chemical treatment [82]; or nuclear transfer [83–85]. The pluripotent nature of the resultant cells makes them a formidable tool for (1) studying embryonic development [86], (2) producing genetically modified animals [87, 88], (3) establishment of in vitro models of genetic diseases [89], and (4) developing new therapies in regenerative medicine [90]. Among the different approaches, OSKM-induced somatic cell reprogramming has become the most widespread technique due to its high reproducibility, applicability to human samples, and simplicity of the process.

OSKM-induced cell reprogramming constitutes an organized sequence of events that starts with the downregulation of somatic cell markers [91]. Then, activation of cell proliferation [92], induction of a metabolic switch from OXPHOS to glycolysis [65], and a mesenchymal-to-epithelial transition (MET) [93, 94] follow. Finally, the process culminates with cellular immortalization [95–100] and upregulation of core pluripotency markers, such *Oct4* or *Nanog* [91, 101]. Also, there is a global erasure of the somatic epigenetic signature during the reprogramming process, which is undertaken by histone-modifying [102–104] and DNA-modifying [105] enzymes. It has been shown that this erasure of the somatic epigenetic marks is increased sequentially cycle after cycle during cell proliferation due to a dilution effect upon cell division [92, 106, 107].

Three seminal studies have demonstrated that the changes undertaken by somatic cells during OSKM-induced cell reprogramming are organized in two consecutive waves [108–110]. The first wave, called stochastic phase, is associated with changes in cell cycle, DNA replication, and MET. The second wave, named deterministic phase, is associated with the total reactivation of the transcriptional core of pluripotency. These studies have revealed that the low efficiency of the process is due to the fact that some of the starting somatic cells are refractory to cell reprogramming and remain trapped as cell intermediates.

**Figure 2:**
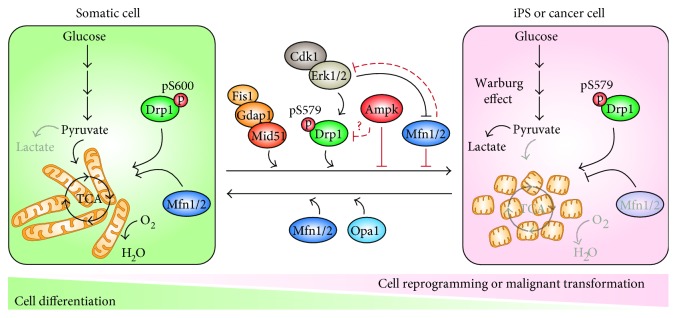
Mitochondrial dynamics in somatic, pluripotent, and cancer cells. Model illustrating the factors involved in the regulation of mitochondrial dynamics and metabolism in somatic, pluripotent (iPS), or cancer cells. The roles played by the indicated factors in favoring (forward arrow) or impairing (reverse arrow) cell reprogramming are also shown.

In contrast to cell differentiation during embryogenesis, it has been suggested that mitochondrial dynamics follows a reverse pathway during cell reprogramming: mitochondria rejuvenate and become fragmented [64, 111], their functionality as energy-producing organelles is reduced [65, 112], and mtDNA replication is decreased [113]. Although it has been suggested that Drp1 does not play a role during OSKM-induced somatic cell reprogramming [114], several reports indicate that this protein plays a key role in this process [78, 115, 116]. In this regard, we have observed an increase in both Drp1 total protein and Drp1-S579 phosphorylation during the stochastic phase of cell reprogramming. During early reprogramming, Erk1 and Erk2 are activated as a consequence of the downregulation of the Dusp6 protein phosphatase. Activated Erk1/2 phosphorylate Drp1-S579, which induces its recruitment to mitochondria and triggers mitochondrial fission during the stochastic phase of cell reprogramming [78] (Figure 2). In addition to Erk1/2, cyclin-dependent kinase 1 (Cdk1) could also participate in the phosphorylation of Drp1-S579 during early cell reprogramming [9]. In fact, it has been observed that the transcriptional factor associated to pluripotency reduced expression protein 1 (REX1) activates *cyclin B* expression in human ES cells. This upregulation activates CDK1/cyclin B complex, leading to an increase in Drp1-S579 phosphorylation and mitochondrial fragmentation. *REX1*-null ES cells show tubular mitochondrial morphology and a decreased self-renewal capacity [116]. Also, pluripotent mouse ES cells lacking a functional *Drp1* gene have been derived by homologous recombination [117] (Figure 2). Although *Drp1* knockout mice show major defects in embryonic development and synapsis formation, *Drp1*-null ES cells maintain pluripotency and self-renewal capacities. *Drp1* knockout cells display a tubular mitochondrial morphology and a lower proliferation rate. Surprisingly, lack of *Drp1* gene does not affect cytokinesis. Given the central role played by Drp1 in mitochondrial fission and that this process is critical to assure an equal distribution of these organelles between the two daughter cells in each cell division, the results obtained by Ishihara and colleagues were puzzling. To circumvent this conundrum, Ishihara and colleagues suggested that unknown mechanical forces could play a role in the uneven segregation of mitochondria between the two daughter cells during cell division.

Conversely to the conventional idea of mitochondrial fission as a mechanism for assuring the equal distribution of mitochondria between the two daughter cells during cytokinesis, a recent report showing that mitochondrial fission also drives the asymmetrical distribution of these organelles during cell division of stem-like cells adds an additional layer of complexity to the physiological roles already ascribed to the fragmentation of these organelles. Interestingly, this asymmetrical distribution of mitochondria depends on the quality of the organelles and whereas aged or deficient organelles are segregated to the more differentiated daughter cell, healthy mitochondria are retained by the resultant stem-like cell upon cytokinesis [118]. Interestingly, this asymmetric segregation of mitochondria contributes to maintain a homogenous and healthy population of stem-like cells, which could be considered as a sort of selfish self-renewal. It would be interesting to investigate whether this unequal segregation of mitochondria takes places under normal and/or pathological conditions in vivo.

Compared to somatic cells, ES cells show low levels of *Mfn1/2* expression [78, 119]. Interestingly, *Mfn1/2* knockout cells display a faster and higher efficiency of cell reprogramming due to an increase of mitochondrial fragmentation and cell proliferation. Also lack of *Mfn1/2* favors Erk1/2 activation, which may improve Drp1-S579 phosphorylation by these MAP-kinases [119]. Furthermore, Erk1/2-mediated phosphorylation of Mfn1 causes its inactivation [37]. Thus, in addition to increase mitochondrial fission through Drp1 phosphorylation [78], Erk1/2 activation during early cell reprogramming may inhibit mitochondrial fusion through Mfn1 phosphorylation (Figure 2).

Recently, it has been described that other proteins involved in mitochondrial fission, such as Mid51 or Gdap1, are also important for cell reprogramming [120]. Downregulation of any of these two proteins reduces cell reprogramming efficiency. Interestingly, *Gdap1* knockout cells displayed a lower reprogramming efficiency due to a defect in triggering mitochondrial fragmentation during the process. The failure to undergo an efficient mitochondrial fission by *Gdap1*-null cells during cell reprogramming induced a DNA damage-independent G2/M arrest (Figure 2).

Important similarities between cell reprogramming and cellular transformation do exist [105]. In this regard, a similar role for mitochondrial fission in tumorigenesis has been proposed [121]. As it happens during cell reprogramming, some cellular transformation processes are associated with MET [122] and changes in mitochondrial morphology: from a tubular network to fragmented and isolated mitochondria. As observed in ES cells, lung [123], gastric [124], breast [125, 126], glioblastoma [127], colorectal [128], neuroblastoma [129], ovarian [130], pancreatic [46], and melanoma [47] cancer cells display high levels of *Drp1* and low amounts of *Mfn1/2* gene expression. Accordingly, inhibition of *Drp1* expression or overexpression of *Mfn1/2* results in a marked reduction of cancer cell proliferation and an increase in spontaneous apoptosis [123–126, 128]. Some other cancer cells are characterized by either a decrease or an increase in *Mfn1/2* and *Fis1* expression levels, respectively [131, 132] (Figure 2).

Furthermore, Drp1 regulation during cellular transformation seems to be similar to that of cell reprogramming. Erk1/2 inhibition in transformed cells decreases Drp1-S579 phosphorylation levels, elongates mitochondria, and reduces cell proliferation and the capability of tumor formation [46, 47]. In lymphoblastic leukemia cells, Erk1/2 triggers Drp1-dependent mitochondrial fission to reduce ROS and enhance glycolysis for protecting cells against chemotherapeutic agents. In this regard, activation of ERK signaling by constitutive expression of a constitutively active K-Ras mutation confers on cells a large degree of phenotypic plasticity that promotes their neoplastic transformation and the acquisition of stem cell-like characteristics [133]. Brain tumor-initiating (BTI) cells display fragmented mitochondria. BTI cells show high levels of DRP1-S579 phosphorylation and targeting DRP1 using RNA interference or pharmacologic inhibition induced apoptosis in BTI cells and inhibited tumor growth [48]. Finally, and similar to *Gdap1*-null cells during cell reprogramming, a knockdown of *Drp1* in lung and breast cancer cells induces a DNA damage-dependent G2/M arrest [134] (Figure 2).

## 4. Mitochondrial Fission and Mitophagy in Cell Reprogramming

Mitochondrial fission is necessary for mitophagy, whereas mitochondrial fusion impairs this mitochondrial-specific form of autophagy [135]. As it has been described in a mitochondrial clearance during cell reprogramming [111, 136, 137], some studies have suggested that mitophagy could be involved in this reduction in mitochondrial mass and therefore play a positive role in the reprogramming process [138, 139]. Accordingly, an induction of autophagy has been shown to increase cell reprogramming efficiency [140] and an early and transitory activation of this process has been observed to take place very early in cell reprogramming to reduce mitochondrial mass [137]. However, new studies have put into question these results. Work by three different laboratories demonstrated that Lc3b/Atg5-dependent autophagy is not responsible for the mitochondrial clearance observed during cell reprogramming [78, 141, 142]. Furthermore, observations by two additional laboratories showed that ES cells have a mitochondrial mass/total protein ratio similar to that of somatic cells [143, 144]. Thus, it seems improbable that an active reduction of mitochondrial mass by mitophagy is taking place during the cell reprogramming process. Altogether, these observations suggest that, beyond its role in the constant turnover of dysfunctional mitochondria, mitophagy seems not necessary for cell reprogramming. It is nonetheless possible that the absolute reduction of mitochondrial mass could be due to an adaptive process to the new culture conditions required to maintain pluripotency [78] or through a Lc3b/Atg5-independent autophagic pathway [141]. Paradoxically, the hypothesis of a passive mitochondrial clearance during successive rounds of cell division would require the generation of new mitochondria to maintain a proper distribution of these organelles between the two daughter cells. In keeping with this idea, it has been reported that mitochondrial biogenesis markers are induced during cell reprogramming [78, 111]. The role of autophagy, in general, and mitophagy, in particular, in the maintenance of pluripotency is poorly understood; however, some studies point that autophagy activation is more important during cell differentiation than during the acquisition of the pluripotent state [145]. Further, chemical activation of Ampk reduced cell reprogramming efficiency [146], and induction of autophagy is associated with an increase in Ampk activation [147]. Interestingly, it has been described that Ampk phosphorylates Drp1-S600, which impairs Drp1 function and mitochondrial fission [55, 148, 149]. Proteomic analysis of Drp1 in mouse ES cells revealed the absence of this posttranslational modification in both mitochondrial and cytosolic fractions of the protein under self-renewal culture conditions [78]. Although it has not been described a role for this phosphorylation in cell reprogramming, it may be similar to that found in cancer cells, where dephosphorylation of Drp1-S600 has been associated to tumor progression [48]. In agreement with this, Ampk activation seems to inhibit cancer progression [150–152]. Further research may shed light into the role of Ampk-mediated Drp1-S600 phosphorylation during the early stages of cell reprogramming (Figure 2).

## 5. Conclusions

In addition to changes in mitochondrial morphology, cell reprogramming induces a metabolic switch: from an oxidative-somatic state to a glycolytic-pluripotent state [153]. This metabolic remodeling presents several similarities with the Warburg effect observed in cancer cells [154]. In fact, there are many processes in which an increase of mitochondrial fission goes along with an activation of glycolysis and a decrease of OXPHOS [155]. The observed changes in both mitochondrial morphology and metabolism seem to be key for cell reprogramming and during the early events of tumorigenesis. Altogether, published data suggest a close parallelism between the stochastic phase of cell reprogramming and cellular transformation [105]. The similarities between both processes reveal that any advance in the control of induced pluripotency will not only help to manage properly this powerful tool for its biomedical application but also to better understand the early events that take place during the development of human malignancies. Interestingly, in vivo cell reprogramming is emerging as an alternative approach to regenerative medicine that does not require cell transplantation [156–159]. Given the importance of mitochondrial dynamics for somatic cell differentiation and dedifferentiation, this mitochondrial process is likely to play a key role in cell fate remodeling during in vivo cell reprogramming. It is therefore possible that the discovery of new techniques to locally modulate mitochondrial dynamics in a specific set of cells, combined with partial in vivo cell reprogramming, will set the grounds for developing novel mitochondria-based therapeutic approaches to improve human welfare.
